# Correction: The metabolomic analysis of five *Mentha* species: cytotoxicity, anti-*Helicobacter* assessment, and the development of polymeric micelles for enhancing the anti-*Helicobacter* activity

**DOI:** 10.1039/d1ra90085d

**Published:** 2021-02-26

**Authors:** Riham O. Bakr, Ahmed F. Tawfike, Heba A. El-Gizawy, Nashwa Tawfik, Usama Ramadan Abdelmohsen, Miada F. Abdelwahab, Walaa A. Alshareef, Sahar M. Fayez, Shereen M. S. El-Mancy, Ahlam M. El-Fishawy, Mostafa A. Abdelkawy, Marwa A. A. Fayed

**Affiliations:** Department of Pharmacognosy, Faculty of Pharmacy, October University for Modern Sciences and Arts (MSA) Giza Egypt; Molecular Discovery Group, Computational and Analytical Science Department, Rothamsted Research Harpenden AL5 2JQ UK; Department of Pharmacognosy, Faculty of Pharmacy, October 6 University Giza Egypt; Department of Pharmacognosy, Faculty of Pharmacy, Helwan University Cairo 11795 Egypt; Department of Pharmacognosy, Faculty of Pharmacy, Deraya University 61111 New Minia Egypt; Department of Pharmacognosy, Faculty of Pharmacy, Minia University 61519 Minia Egypt usama.ramadan@mu.edu.eg +2-86-2347759; Department of Microbiology and Immunology, Faculty of Pharmacy, October 6 University Giza Egypt; Department of Pharmaceutics and Industrial Pharmacy, Faculty of Pharmacy, October 6 University Giza Egypt; Department of Pharmacognosy, Faculty of Pharmacy, Cairo University 11562 Cairo Egypt; Department of Pharmacognosy, Faculty of Pharmacy, University of Sadat City Sadat 32897 Egypt

## Abstract

Correction for ‘The metabolomic analysis of five *Mentha* species: cytotoxicity, anti-*Helicobacter* assessment, and the development of polymeric micelles for enhancing the anti-*Helicobacter* activity’ by Riham O. Bakr *et al.*, *RSC Adv.*, 2021, **11**, 7318–7330, DOI: 10.1039/D0RA09334C.

The authors regret that the name and affiliations were incorrectly shown for Ahmed F. Tawfike in the original manuscript. In addition, it was not shown that Riham O. Bakr and Ahmed F. Tawfike contributed equally. The corrected author list and affiliations are given here.

The Royal Society of Chemistry apologises for these errors and any consequent inconvenience to authors and readers.

## Supplementary Material

